# Low-Loss Multimode Waveguide Bends with Direct Laser Writing in Polymer

**DOI:** 10.3390/mi16121361

**Published:** 2025-11-29

**Authors:** Tigran Baghdasaryan, Neshteh Kourian, Mushegh Rafayelyan, Tatevik Sarukhanyan

**Affiliations:** 1 Vrije Universiteit Brussel, Department of Applied Physics and Photonics, Brussels Photonics, Pleinlaan 2, 1050 Brussel, Belgium; 2 PhotonicsAI Lab, Institute of Physics, Yerevan State University, 1 Alex Manoogian, Yerevan 0025, Armenia; neshteh.kourian@ysu.am (N.K.); mushegh.rafayelyan@ysu.am (M.R.); tatevik.sarukhanyan@ysu.am (T.S.)

**Keywords:** multimode waveguides, Bézier shape bends, direct laser writing, 2 photon polymerization

## Abstract

Waveguide bends are critical components for compact routing in integrated photonic circuits, yet their design in air-clad polymer waveguides fabricated by two-photon polymerization direct laser writing (2PP-DLW) is challenging due to multimode behavior. We address this by systematically modeling Bézier-shaped 90° bends and S-bends using a variational FDTD solver, exploring bend span, curvature, and waveguide dimensions. Our results show that smaller waveguides (widths 2–4 µm) and lower Bézier parameters (B = 0–0.2) consistently yield superior performance, enabling sharper bends with minimal loss. For 90° bends, spans as small as 20–30 µm achieve near-unity transmission, while for S-bends, aspect ratios below 1 are feasible, allowing highly compact layouts. Although all configurations transmit energy to the fundamental mode, wider waveguides exhibit stronger higher-order mode excitation and greater sensitivity to fabrication imperfections. Smaller waveguides reduce these effects but approach the resolution limits of 2PP-DLW. Thus, a 2 µm wide waveguide represents an optimal compromise between fabrication feasibility and optical performance. Experimental demonstrations confirm the practicality of these design rules, illustrating trends predicted by simulations. These findings establish clear guidelines for designing low-loss, space-efficient 3D photonic circuits and highlight the critical role of simulation-driven optimization in fully exploiting 2PP-DLW technology, while providing deeper insight for future device architectures.

## 1. Introduction

Direct laser writing using a two-photon polymerization approach is a growing field in manufacturing technologies that allows for rapid and maskless fabrication of complex 3D geometries [1,2,3]. By tightly focusing a laser beam in specially developed photoresists and by taking advantage of multiphoton absorption polymerization mechanism, fabrication resolutions smaller than the diffraction limit of the focused laser beam are within reach. As a result, the fabrication of wavelength-scale photonic structures is possible nowadays [4,5,6,7,8,9,10,11,12,13,14,15,16]. Among others, the fabrication of waveguiding structures is feasible with direct laser writing, and traditional fields, such as optical communication and optical sensing, are making use of this development [9,11]. There have been many reports of 3D-printed devices based on polymer waveguides, tapers, resonators, gratings, couplers, etc. Over time, the complexity of such devices increases as well [15,17,18]. We can address such structures by a common term “3D photonic integrated circuits” (3D PICs) in analogy to planar photonic integrated circuits, as they share a common definition.

The majority of efforts in the domain focus on developing devices for certain photonic applications, while a thorough study of the separate components that such a device uses is often missing. We believe that significant efforts should be put into optimizing such building blocks of 3D-printed PICs, as it will accelerate the development of the domain, offering optimized and ready-to-use solutions for more complex device architectures. Despite the significant developments and accumulated knowledge of optical components for planar PICs, many design and fabrication rules should be revisited for 3D polymer PICs. Among the features that distinguish 3D polymer PICs, we can mention the material properties and refractive index with mostly air cladding architecture, the design freedom on waveguide shape and size, as well as the maskless fabrication approach of point-by-point, line-by-line, or plane-by-plane polymerization with sub-micrometer resolutions. As a result, dimensions, surface roughness, geometries, and fabrication speeds of 3D polymer optical components are very different from Si- or InP-based components.

In our previous research, we have already studied the design and fabrication aspects of fiber-coupled waveguides, tapers, and S-bends [10,11,18,19]. We have shown that for square-shaped waveguides, coupling loss to standard single-mode optical fibers can be below 0.25 dB, while propagation losses in 14 × 14 µm cross-section waveguides are in the range of 1.1 dB/mm [11]. For tapers, we have studied parabolic shapes that allow for lossless transitions to different waveguide sizes and experimentally demonstrated a nearly lossless taper from 14 µm to 2 µm wide waveguides with a length as short as 55 µm as compared to linear tapers [10,11]. Using these building blocks, we have demonstrated on-fiber tip mode conversion tapers facilitating fiber-to-chip coupling, and ultracompact splitters for single-core to multi-core fiber interconnections that featured losses less than 3 dB [18].

While tapers are essential for mode conversion and efficient coupling between waveguides of varying dimensions, another equally critical component in integrated photonic circuits is the waveguide bend. Bends facilitate the redirection of light from its original propagation path, enabling compact routing and the realization of complex circuit layouts with minimal loss and mode distortion.

Indeed, waveguide bends have been a subject of extensive research since the early development of photonic integrated circuits [20]. Despite decades of progress, recent studies continue to explore novel bend geometries aimed at achieving lower losses and more compact configurations [21,22,23,24,25,26,27,28]. While earlier research focused on bends based on circular arc sections, further research extended to looking into specific bend curve functions, with Bézier and Euler bends being the focus. Bends based on subwavelength-structured waveguides or metawaveguides also showed promising potential [29,30]; however, their application in 3D-printed waveguides remains challenging due to the limited resolution of 2PP-DLW technology compared to lithography-based nanoprinting used for PICs, and this can be considered as a topic for future research. Particular attention has been directed toward multimode waveguide bends as well [24,26,31], where the design objectives can be twofold: first, to ensure that the fundamental mode propagates with minimal loss; and second, to maintain low-loss transmission for higher-order modes as well. These considerations and design variabilities are crucial for applications involving mode-division multiplexing and multimode photonic signal processing.

Although bends are critical components for 3D-printed PICs, there is limited insight into their design considerations beyond our previous reports [11,19]. In most studies, including ours, bends have been addressed as part of a broader component design task, with little discussion of their specific design principles [9,15,32,33,34,35,36]. References to bending radius in many works typically suggest circular-arc-based designs, underscoring the need for greater focus on optimizing bend geometries for more effective and efficient designs. Only [32] examined bend design in greater detail, investigating both sigmoid and circular-arc shapes within their approach of free-form waveguide interconnects. However, the design considerations presented there are tailored to the authors’ system-level approach and are not generic. To the best of our knowledge, no dedicated study has focused exclusively on the bending challenges of 3D-printed polymer waveguides. This work represents the first systematic investigation devoted solely to 3D-printed waveguide bends, delivering critical insights into their design aspects and introducing a comprehensive modeling framework as the core contribution to establish clear design rules for their fabrication. Here, we examine multimode propagation in detail, present an extended parameter study, and define practical guidelines for low-loss bend design and fabrication. Furthermore, 90° bends are introduced for the first time in this context, alongside a systematic analysis of S-bends, enabling sharper routing and more compact layouts in 3D photonic integrated circuits.

The remainder of this paper is organized as follows: Section 2 introduces the design considerations for fiber-coupled polymer waveguides and highlights the impact of high-index contrast on multimode behavior. Section 3 presents the core modeling results: first, a detailed analysis of 90° bends (Section 3.1), followed by the study of S-bends and their parameter dependencies (Section 3.2). Section 3.3 provides experimental demonstrations illustrating the practical feasibility of the proposed design rules. Finally, Section 4 discusses the implications of these findings for future 3D photonic integrated circuits and outlines directions for further optimization.

## 2. Fiber-Coupled Waveguides

**Figure 1:**
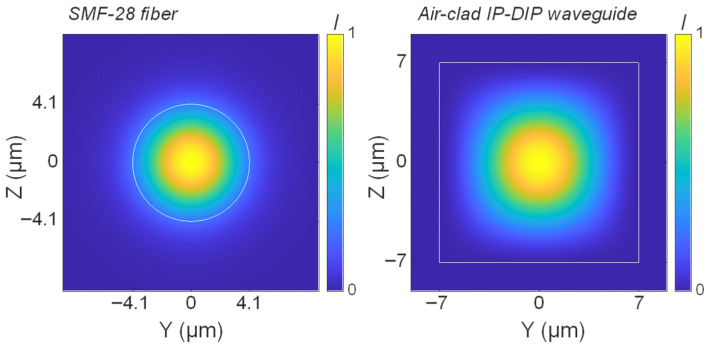
Mode intensity distributions simulated using ANSYS Lumerical MODE software for a standard telecom-grade SMF-28 fiber (**left**) and a mode-matched polymer waveguide (**right**) with a square cross-section of 14 × 14 µm^2^, fabricated in IP-DIP material.

In this study, we investigate polymer waveguides that can be fabricated using IP-DIP photoresist [37], a material optimized for high-resolution 3D printing with the Nanoscribe system. The analysis was conducted at the telecom wavelength of 1550 nm, for which the refractive index of polymerized IP-DIP is 1.53. Optical fibers remain the primary method for coupling light into and out of photonic devices, as well as for interconnection applications. Therefore, we begin by examining waveguide geometries that enable efficient coupling with a standard telecom-grade single-mode fiber (SMF-28, Corning Inc., Corning, NY, USA), while minimizing insertion losses. As we have demonstrated in [11], a square cross-section, air-clad IP-DIP waveguide, with dimensions of 14 × 14 µm^2^, offers minimal coupling losses with SMF-28 fiber. Figure 1 presents the mode field intensity of the SMF-28 fiber alongside the mode profile of the 14 × 14 µm^2^ cross-section polymer waveguide at 1550 nm. Mode-overlap simulations performed using the ANSYS (2023 R2.2) Lumerical MODE indicate a power overlap of 0.96 between the fiber and the waveguide modes, corresponding to an insertion loss of approximately 0.2 dB.

It is worth noting that a substantial dimensional mismatch exists between the fiber core and the mode-matched polymer waveguide. While the SMF-28 fiber has a core diameter of 8.2 µm, the mode-matched IP-DIP waveguide is considerably larger. This discrepancy arises from the difference in refractive index contrast between the core and cladding of the two configurations. SMF-28 features a low core-clad index contrast of approximately 0.005, whereas the IP-DIP waveguide exhibits a much higher contrast of about 0.53. As a result, the fiber supports only a single mode at 1550 nm, while the IP-DIP waveguide is highly multimode. Consequently, careful design considerations are required to make sure that light is being guided within the fundamental mode. On the other hand, the larger refractive index contrast of air-clad polymer waveguides allows for sharper bends, as will be discussed later.

From a design perspective, employing a polymer waveguide that is mode-matched to a standard SMF-28 fiber represents an intuitive choice for light guiding and routing. However, this configuration imposes constraints on bend tolerance, particularly in applications where confinement to the fundamental mode is critical. Larger waveguides support a greater number of modes, and their highly multimoded nature leads to increased coupling into higher-order modes, even under moderate bending conditions. This behavior will be demonstrated in Section 3 through simulations of 90° bends. To mitigate this issue, we have proposed tapering the waveguide to a smaller cross-section. This reduces the number of supported modes and increases the effective index separation between higher-order modes. A larger index gap helps suppress intermodal coupling, enabling tighter bends while maintaining low-loss transmission of the fundamental mode in few-mode waveguides.

To explore this behavior, we studied waveguide bending in a single plane using rectangular waveguides with a fixed height of 14 µm (Z-span) and varying width (Y-span). Throughout this work, we consider bends in the XY plane, with the input waveguide cross-section defined in the YZ plane. Bending in the XY plane primarily excites modes that differ in their Y-profile, particularly those with opposite parity in the Y-direction. Modes that differ only in the Z-profile or share the same Y-parity are not efficiently coupled by such bends.

**Figure 2:**
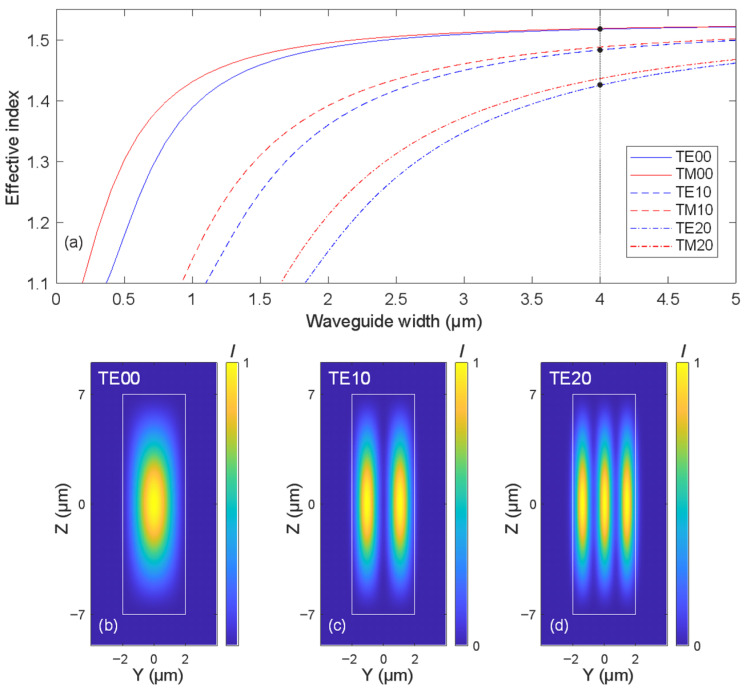
(**a**) Simulated effective refractive indices of TE and TM modes at 1550 nm wavelength as a function of waveguide width for a rectangular polymer waveguide with fixed height of 14 µm. The plot includes TE00, TM00, TE10, TM10, TE20, and TM20 modes, with waveguide widths ranging from 0 to 5 µm. Bottom: Mode intensity distributions for (**b**) TE00, (**c**) TE10, and (**d**) TE20 modes, shown in the YZ plane.

Figure 2a presents simulation results for the effective refractive index of a rectangular polymer waveguide with varying widths. The analysis focuses on the three highest-order transverse electric modes: TE/TM00, TE/TM10, and TE/TM20. The corresponding mode intensity distributions for TE modes are shown in Figure 2b, Figure 2c, and Figure 2d, respectively. As the waveguide width decreases, the separation between the effective indices of the adjacent modes increases. This trend is observed not only among higher-order TE modes but also between the TE and TM modes of the same order. The increased index spacing contributes to reduced intermodal coupling, which is advantageous for maintaining mode purity in compact photonic circuits. Similar trends apply when considering waveguides with a reduced height (Z-extent), with the distinction that the effective indices become lower and mode cutoffs slightly shift.

We also clearly see that for the smaller waveguide dimensions, polarization-dependent behavior becomes more pronounced due to increased asymmetry and stronger confinement. In such cases, particular attention must be given to polarization-induced birefringence and mode coupling, which can significantly affect device performance and must be carefully managed in design and fabrication.

## 3. Results

### 3.1. 90-Degree Waveguide Bends

**Figure 3:**
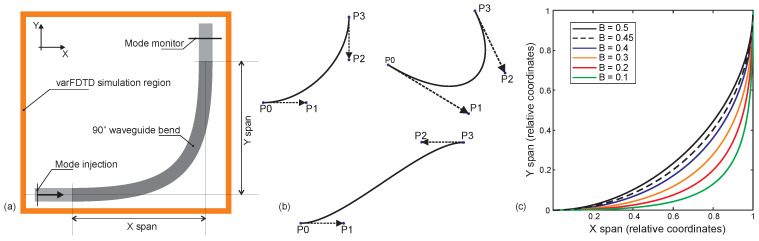
(**a**) Schematic of the varFDTD simulation outline for 90° waveguide bends, showing the simulation region, mode injection point, and mode monitor. (**b**) Bézier curve configurations defined by P_0_, P_1_, P_2_, and P_3_ control points. (**c**) Geometries of 90° waveguide bends for Bézier parameters ranging from 0.1 to 0.5.

In this section, we present the results of a numerical investigation into 90-degree bends in polymer waveguides with rectangular cross-sections. The waveguides considered are compatible with fabrication using the Nanoscribe system in IP-DIP photoresist and have been demonstrated in [11]. The refractive index of polymerized IP-DIP has been characterized and reported in the literature across a broad wavelength range, and these values were used in our simulations [38]. Mode propagation in both straight and bent waveguide sections was modeled using the varFDTD solver in Ansys Lumerical MODE Solutions. This solver enables the reduction of three-dimensional propagation problems into two-dimensional simulations by first computing the effective refractive indices of the guided modes in the waveguide cross-section, followed by simulating their propagation in the transverse plane. Figure 3a illustrates the simulation domain used for analyzing 90-degree waveguide bends. The structure consists of straight input and output waveguide sections connected by a bent region. Fundamental mode is launched into the input section using the mode source. At the output, the simulation evaluates the fraction of optical power coupled back into the fundamental mode, allowing assessment of bend-induced mode distortion and loss. We used perfectly matched layer (PML) boundary conditions with an automated non-uniform mesh setting at mesh accuracy level 3, which reduced the simulation time to less than one minute for a single S-bend. This fast simulation capability enabled us to perform an extensive study using a standard desktop computer.

To design 90-degree waveguide bends, we employed Bézier-shaped geometries to define the curved transition region. Bézier curves, originally developed for smooth transitions in computer graphics, are now widely used in photonic design due to their flexibility and ability to support adiabatic mode transitions [39]. Additionally, Bézier transitions are straightforward to implement within the ANSYS Lumerical simulation environment. Each bend was constructed using a Bézier curve defined by four control points: P0 and P3 specify the start and end points of the bend, while P1 and P2 control the curvature. Examples of Bézier-shaped curves are shown in Figure 3b. To ensure proper alignment with the input and output waveguide sections, P1 and P2 were placed along the axes of the respective waveguides. The shape of the bend is governed by the Bézier parameter B, which determines the position of P1 according to the relation P0P1 = (bend span) × (1 − B). Due to symmetry, the same relation applies to P3P2. A value of B = 0.5 places P1 and P2 at the middle of the bend span.

Figure 3c shows the geometries of 90-degree waveguide bends constructed using Bézier curves with parameters ranging from 0.1 to 0.5. Each bend is defined by four control points, with the Bézier parameter B controlling the curvature profile. Notably, the bend corresponding to B = 0.45 closely approximates a circular arc and is therefore indicated with a dotted line. Bends with smaller Bézier values exhibit smoother transitions near the input and output ends and sharper curvature near the center of the bend.

**Figure 4:**
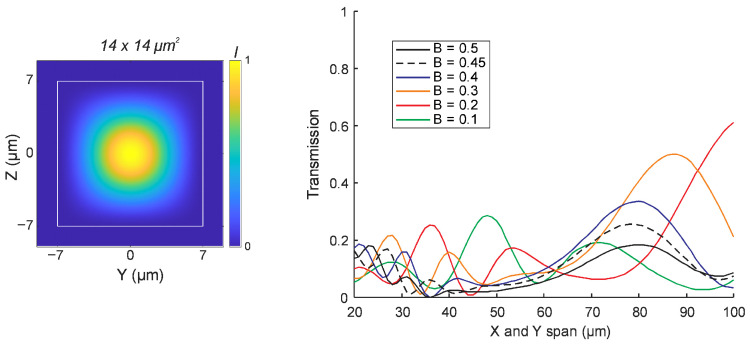
**Left**: Spatial intensity distribution of the fundamental mode in a 14 × 14 µm^2^ cross-section IP-DIP waveguide. **Right**: Simulated transmission through 90° waveguide bends as a function of bend span (X and Y) for various Bézier parameters (B = 0.1 to 0.5). Each curve represents a different B value, with B = 0.45 corresponding to a circular arc indicated by a dotted line. Simulations were performed at a wavelength of 1550 nm using a refractive index of 1.53 for the polymerized IP-DIP.

Using the modeling approach described above, we first investigated the transmission of the fundamental mode through a 90-degree bend in a mode-matched IP-DIP waveguide with a square cross-section of 14 × 14 µm^2^. In all cases, the bend geometry was defined with equal X and Y spans. The results are presented in Figure 4, which shows the transmission performance for various bend spans ranging from 20 µm to 100 µm, and for different Bézier parameters. Light was injected in the fundamental mode, and the transmitted power in the same mode was monitored at the output. Given the low polarization dependence, results are presented for TE polarization only.

None of the simulated bend configurations resulted in a low-loss transmission of the fundamental mode. The highest transmission observed was approximately 0.6, corresponding to a Bézier parameter of B = 0.2 and a bend span of 100 µm. The circular arc bend, represented by B = 0.45, did not exceed a transmission of 0.3. For B = 0.3, a peak transmission of around 0.5 was recorded for the bend span of 88 µm. These results indicate that achieving low-loss transmission of the fundamental mode in 90-degree bends is challenging in highly multimode waveguides. Moreover, sharp bends with compact footprints (X and Y span below 100 µm) do not yield efficient transmission within the range of Bézier geometries considered in this study.

**Figure 5:**
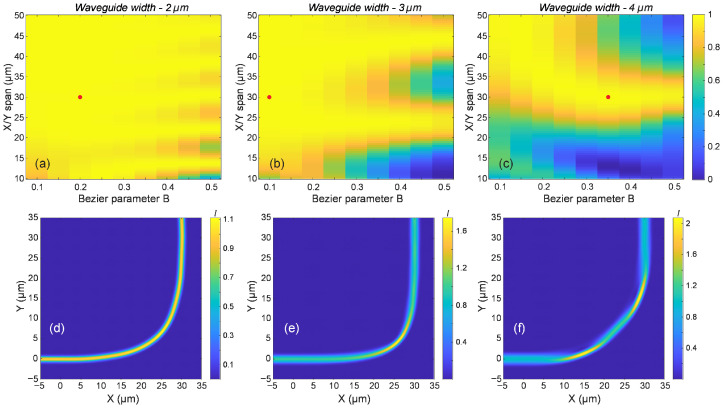
(**a**–**c**) Simulated transmission of the fundamental mode through 90° waveguide bends for a range of Bézier parameters and bend spans, for waveguide widths of (**a**) 2 µm, (**b**) 3 µm, and (**c**) 4 µm. Red dots indicate selected design points with high transmission, with the corresponding spatial intensity distributions in the bend regions, as shown in (**d**–**f**) for the designs marked as (**a**), (**b**), and (**c**), respectively.

As the waveguide dimensions decrease and the separation between the effective indices of higher-order modes increases, sharper bends with adiabatic transmission of the fundamental mode become feasible. Figure 5 illustrates the results for waveguide widths of 4 µm, 3 µm, and 2 µm. Fabricating waveguides with dimensions below 2 µm using two-photon polymerization direct laser writing (2PP-DLW) becomes increasingly challenging. At these scales, the dimensions approach the resolution limits of the technique, and the scattering losses due to waveguide wall surface roughness become substantial as well. We have previously demonstrated that the transition from a fiber-matched waveguide to such waveguides can be achieved through tapering [11], and that employing parabolic-shaped tapers enables shorter transition regions while maintaining adiabatic behavior [40].

Figure 5a–c present two-dimensional colormaps where both the X and Y spans of the bend are varied equally, and the transmission of the fundamental mode is simulated. Regions of high transmission are indicated in yellow, allowing for the immediate visual identification of efficient bend configurations. Notably, sharp bends with spans below 50 µm can yield transmission values approaching unity. While careful design is required to achieve high transmission for the 4 µm waveguide, the 2 µm configuration exhibits a broad parameter space with consistently high transmission, slightly lower efficiency regions around 10 µm spans, and higher Bézier parameters. In general, lower values of the Bézier parameter lead to improved performance. An exception is observed for the 4 µm waveguide, where sharper bends with spans in the 25–30 µm range benefit from a Bézier parameter closer to 0.3. Achieving low-loss transmission through sharper bends with spans below 25 µm remains challenging for waveguides with widths of 4 µm and 3 µm. However, for 2 µm wide waveguides, careful selection of the Bézier parameter can enable transmission efficiencies approaching unity.

Figure 5d–f illustrate the intensity distribution in waveguide bends, with the corresponding design parameters indicated by red dots in the figures above. All configurations exhibit near-unity transmission and a bend span of 30 µm, but differ in Bézier parameters and waveguide widths. Figure 5d presents the results for a 90° bend with a 2 µm wide waveguide and a Bézier parameter B = 0.2. The intensity distribution remains nearly uniform along the bend, suggesting minimal excitation of higher-order modes during beam propagation. Figure 5e shows a bend with a 3 µm wide waveguide and B = 0.1. While the optical power is clearly confined to the fundamental mode at the output, the central bending region exhibits varying intensity and values exceeding 1. This indicates the presence of higher-order mode excitation. A more pronounced effect is observed in Figure 5f, which corresponds to a 4 µm wide waveguide with B = 0.35. Here, periodic energy transfer between modes and localized regions of high intensity are clearly visible, highlighting significant multimode interactions.

Although all three configurations successfully transmit energy to the fundamental mode at the output, wider waveguides, with their reduced effective index separation and increased multimode nature, are more susceptible to higher-order mode excitation. Fabrication imperfections can further exacerbate this effect in larger waveguides. In contrast, narrower waveguides, which support fewer modes and exhibit greater effective index separation, are less prone to such disturbances. However, fabricating smaller waveguides poses challenges due to the resolution limits of the 2PP-DLW technology. Based on these observations, we consider the 2 µm wide waveguide a suitable compromise between fabrication feasibility and optical performance, despite its few-mode nature.

### 3.2. Waveguide S-Shaped Bends

**Figure 6:**
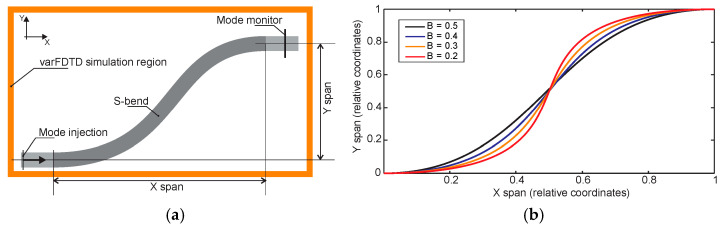
(**a**) Schematic of the varFDTD simulation outline for waveguide S-bends, showing the simulation region, mode injection point, and mode monitor. (**b**) Geometries of waveguide S-bends for Bézier parameters ranging from 0.2 to 0.5.

We now turn our attention to S-shaped bends in polymer waveguides or simply S-bends. A similar simulation approach is employed as in the previous section, allowing us to evaluate mode propagation and transmission characteristics under varying geometric parameters. Figure 6a illustrates the simulation domain used for analyzing waveguide S-bends. Here as well, mode propagation in both straight and bent waveguide sections was modeled using the varFDTD solver in Ansys Lumerical MODE Solutions. The structure consists of straight input and output waveguide sections connected by a bent region defined by a Bézier curve. Light is launched into the input section using the fundamental mode of the waveguide. The S-bend is defined by X and Y spans, and the general goal is to identify the smallest length (X span) for the given waveguide axis offset (Y span). At the output, the simulation evaluates the fraction of optical power coupled back into the corresponding fundamental mode, enabling the assessment of bend-induced loss. Figure 6b shows the geometries of waveguide S-bends constructed using Bézier curves with parameters ranging from 0.2 to 0.5. Each bend is defined by four control points, with the Bézier parameter B controlling the curvature profile and overall shape of the bend as explained in Section 3.1. In this section, we target the waveguide S-bends for a fixed 90 µm offset for 1550 nm wavelength, and we extend the study by examining the influence of input polarization. While the choice of lateral offset could have been different, the overall methodology, observed trends, and key conclusions remain unaffected.

**Figure 7:**
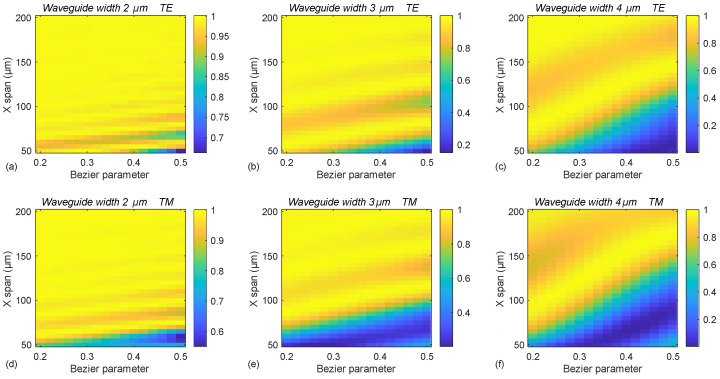
(**a**–**f**) Simulated transmission of the fundamental mode through waveguide S-bends designed to achieve a 90 µm lateral offset, evaluated across varying Bézier parameters, S-bend lengths (X spans), and two orthogonal polarizations. Results are shown for waveguide widths of 2 µm in (**a**,**d**), 3 µm in (**b**,**e**), and 4 µm in (**c**,**f**).

Figure 7 presents simulation results for air-clad IP-DIP waveguides with a fixed height of 14 µm and widths of 2, 3, and 4 µm. For a constant lateral offset of 90 µm, the simulations demonstrate that near-unity transmission of the fundamental mode is achievable for all the considered waveguide sizes and input polarizations. Within the examined S-bend lengths (X spans) ranging from 50 to 200 µm, the widest transmission range is observed for waveguides with a 2 µm width, where even the least favorable configurations yield transmission values exceeding 0.5.

As the waveguide width increases to 3 and 4 µm, the parameter space associated with lower transmission (indicated by the blue regions) expands, with the minimum transmission values approaching 0.1 for a Bézier parameter of 0.5 and shorter S-bend lengths. A general trend emerges, indicating that lower Bézier parameters tend to result in better transmission performance. However, across all configurations, oscillatory behavior in transmission is observed as the X span varies for a fixed Bézier parameter. This highlights the importance of careful design parameter selection to ensure adiabatic transmission of the fundamental mode.

For the two orthogonal polarizations, the transmission behavior is nearly identical, though the patterns are shifted along the X-span axis. This suggests that for polarization-independent performance, designs must be selected such that both polarizations yield high transmission simultaneously. Most notably, the results show that to achieve the target 90 µm vertical offset (Y shift), the required S-bend length (X span) can be shorter than 90 µm. This indicates the feasibility of designing S-bends with an aspect ratio less than 1, which is a significant characteristic of air-clad IP-DIP waveguides. Such compact bend geometries highlight the potential of this platform for highly integrated and space-efficient photonic circuit designs.

**Figure 8:**
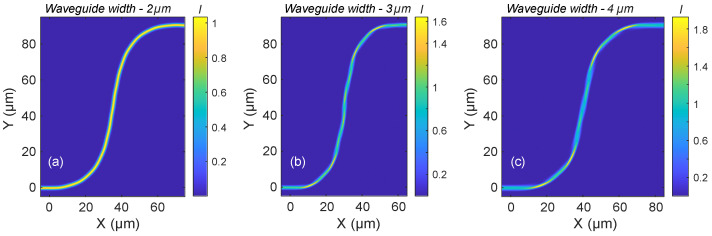
Intensity distributions in selected S-bend waveguides exhibiting greater than 99% transmission of the fundamental mode at a wavelength of 1550 nm and TE polarization, enabling a 90 µm lateral offset. (**a**) Waveguide width of 2 µm, Bézier parameter 0.2, and length of 70 µm; (**b**) waveguide width of 3 µm, Bézier parameter 0.2, and length of 60 µm; (**c**) waveguide width of 4 µm, Bézier parameter 0.2, and length of 80 µm.

Figure 8a–c illustrate the intensity distributions in selected waveguide S-bends that exhibit nearly adiabatic propagation of the fundamental mode at a wavelength of 1550 nm for TE polarization. Based on the findings from the broader parameter study in Figure 7, all examples shown here use the lowest Bézier parameter considered (0.2), which consistently yielded high transmission.

Similar to the behavior observed in 90° bends, these S-bends also exhibit multimode propagation characteristics, with noticeable cross-coupling between modes during light propagation. For the 2 µm wide waveguide (Figure 8a), the peak intensity remains close to 1, and the distribution is nearly uniform along the bend, indicating minimal mode distortion. In contrast, the 3 µm wide waveguide (Figure 8b) shows a peak intensity approaching 1.6, with more pronounced variations in the intensity profile. The 4 µm wide waveguide (Figure 8c) exhibits even stronger intensity fluctuations, with peak values reaching approximately 1.8.

Despite these differences in internal mode dynamics, all three configurations result in clean excitation of the fundamental mode at the output, confirming that high transmission and mode purity can be maintained even in compact S-bend geometries.

### 3.3. Experimental Demonstrations of Waveguide S-Bends in IP-DIP Polymer

Although the primary focus of this manuscript is on modeling and the formulation of design rules for multimode waveguide bends fabricated with high-resolution 3D-printing technology, we also include experimental demonstrations to showcase their practical implementation. These examples, drawn from our previous and ongoing work, showcase waveguide bends fabricated using 2PP-DLW technology in accordance with the design principles presented above. We should emphasize that the results below do not serve as validation, but rather illustrate the practical feasibility of implementing the proposed design rules and highlight considerations for experimental optimization.

**Figure 9:**
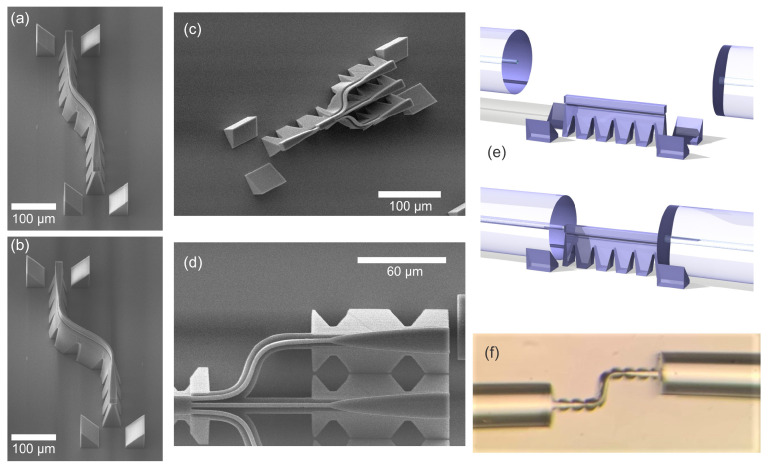
SEM images of 3D-printed waveguide components fabricated using 2PP-DLW technology. (**a**,**b**) show S-bends with lateral offsets of 50 µm and 100 µm, respectively, used for transmission characterization. (**c**,**d**) illustrate a 1 × 3 in-plane splitter incorporating Bézier-shaped S-bends (B = 0.2) with a 31.5 µm offset for multi-core fiber coupling. (**e**) presents an ultracompact 1 × 4 3D splitter [18] featuring S-bends optimized for a 32 µm offset, enabling an overall footprint of 180 µm and insertion loss of less than 3 dB per channel. (**e**) Illustration of the transmission measurement approach with two SMF-28 fibers aligned on the v-grooves for butt-coupling with the waveguide component under study. (**f**) A microscope image of a waveguide with an S-bend and two butt-coupled fibers during the transmission measurement process.

Figure 9a,b show SEM images of S-bends fabricated in a configuration commonly used for transmission characterization. The characterization method, described in detail in [11], employs a suspended waveguide structure supported by narrow ridges to elevate the component above the substrate. This elevation facilitates efficient butt-coupling of optical fibers, while integrated V-grooves on both sides enable rapid and accurate fiber alignment. Figure 9f schematically illustrates the transmission measurement procedure, where two fibers for input and output coupling are positioned in grooves on opposite sides of the waveguide component to enable butt-coupling. Figure 9f shows a microscope image of the measurement setup for S-bends. Based on numerical and experimental estimates from our previous work, this butt-coupling configuration with an air gap introduces approximately a 0.2 dB loss per interface. Furthermore, parabolic tapers are implemented at the input and output to ensure adiabatic mode transformation between the optical fiber and the waveguide cross-section.

Figure 8a shows an S-bend providing a 50 µm lateral offset of the optical axis with a Bézier parameter of 0.5. The bent waveguides have a cross-section of 14 × 4 µm^2^. Transmission measurements, here and in subsequent sections, were performed using an unpolarized amplified spontaneous emission (ASE) source covering the C + L telecom bands. For the 50 µm offset design, S-bends with lengths of 140 µm, 160 µm, and 180 µm were fabricated. The measured transmissions, normalized to butt-coupled optical fibers, were 57% and 60% for two 140 µm-long S-bends, 67% and 68% for two 160 µm-long S-bends, and 58% and 55% for two 180 µm-long structures, respectively.

Figure 9b shows an S-bend in a similar configuration (with Bézier parameter 0.5), providing a 100 µm lateral offset using an identical 14 × 4 µm^2^ cross-section waveguide. For the two 120 µm-long S-bends, we measured a 29% and 32% transmission, while for longer S-bends these values were higher. For the two 140 µm-long S-bends, we measured a 61% and 63% transmission, while for the 160 µm-long S-bends these values were 56% and 60%.

For straight waveguides with integrated tapers, transmission typically reaches about 80%. In comparison, achieving overall transmission in the range of 65–70% for S-bends indicates that low-loss propagation is feasible. As a key takeaway, we emphasize that, alongside numerical modeling, experimental optimization remains essential to ensure minimal loss through S-bends. Figure 9c–e illustrate splitters designed for single-core to multi-core fiber interconnects. The splitter shown in Figure 9c,d implements 1 × 3 in-plane splitting and incorporates S-bends identical to those analyzed earlier in this work. The main splitting element is a multimode interference (MMI) coupler, featuring input and output waveguides with a cross-section of 14 × 2 µm^2^. The design objective was to interface a standard SMF-28 fiber on one side and distribute light to three cores of a commercially available Fibercore seven-core single-mode fiber (SM-7C1500, Fibercore Limited, Hampshire, UK). To match the pitch of the multi-core fiber, the target lateral shift for each S-bend was 31.5 µm. For this purpose, a Bézier-shaped bend with B = 0.2 was selected, resulting in an S-bend length of 46 µm. Ideally, the splitter would deliver 33.3% transmission to each output waveguide; experimentally, we measured approximately 23% transmission for the central core and about 15% for the side cores. These results indicate that total propagation losses through the component remain below 50% or 3 dB. Despite the smaller waveguide dimensions, the S-bends exhibited low loss, while the compact footprint of the splitter represents a significant advantage.

### 3.4. 1 × 4 Single-Core to Multi-Core Fiber Splitter Featuring 3D S-Bends

**Figure 10:**
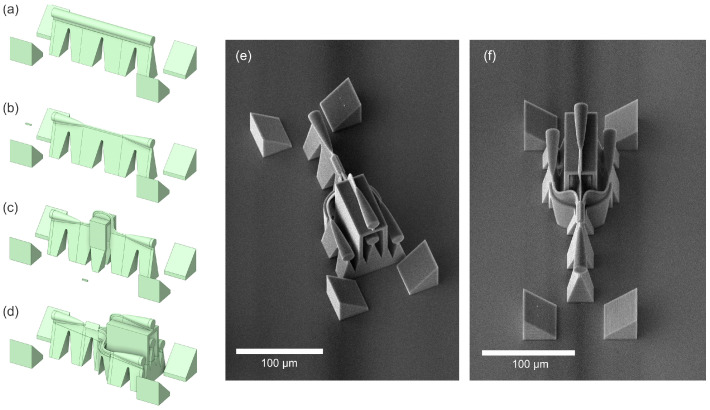
Design evolution and fabricated structures of the 1 × 4 3D splitter. (**a**) Straight waveguide with a 15 µm diameter. (**b**) Waveguide with two tapers reducing the central section to a 2 µm diameter. (**c**) Waveguide with two S-bends introducing a 32 µm lateral offset. (**d**) Complete design of the 3D-splitter incorporating a triangular cross-section MMI. (**e**,**f**) SEM images of the fabricated 3D splitters with the integrated alignment features.

Last but not least, Figure 10e,f present a 3D splitter designed for 1 × 4 splitting in multi-core fiber interconnects, which we have previously reported in [18]. This is a perfect example of when a design methodology that we have presented previously is fully exploited for an application showcasing the full 3D flexibility of the 2PP-DLW approach. The S-bends used in this demonstrator were optimized with 2D simulations, and a further three of such bends have been designed in 3D space by rotating them along the input axis. The ultracompact S-bends were optimized through extensive simulations to achieve a lateral offset of 32 µm for each branch. Initially, square 2 × 2 µm^2^ waveguides were modeled using a 2D variational FDTD solver to explore the Bézier parameters (B = 0–0.5) and lengths from 20 to 50 µm. The best performance was observed for B = 0, yielding insertion losses below −0.1 dB across the C + L band. Subsequent 3D simulations for circular 2 µm diameter waveguides confirmed an optimal S-bend length of 36 µm with near unity transmission. These findings enabled the fabrication of a splitter with an overall length of 180 µm and measured insertion loss of less than 3 dB per channel, demonstrating the potential of 2PP-DLW technology for rapid, low-loss, and highly compact multi-core fiber coupling solutions in full 3D geometry.

During the experimental optimization of the splitter, we analyzed the loss contributions of individual building-block components that are relevant to this work and have not been previously reported. Figure 10a–d illustrate progressively more complex designs, all with the same length of 180 µm and fabricated under identical conditions (see Methods section in [18] for details). The first design (Figure 10a) consists of a straight waveguide with a diameter of 15 µm. Next, we introduced tapers on both ends, reducing the diameter to 2 µm in the middle section (Figure 10b). Subsequently, two S-bends were added, offsetting the waveguide axis by 32 µm before returning to the original level. Finally, we fabricated the complete 3D splitter by incorporating the triangular cross-section MMI. Each component was printed in four replicas and characterized using an unpolarized ASE source spanning wavelengths from 1525 nm to 1610 nm. Measurement was achieved by the approach shown in Figure 9e, using SMF-28 fibers except for the splitter, which was characterized with seven-core fibers. The reference measurement was obtained by butt-coupling two SMF-28 fibers.

For the straight waveguides (Figure 10a), we measured an average transmission of 87.8 ± 0.7% (standard deviation calculated for four components), which is close to the theoretical prediction of 0.2 dB or 4.5% loss per facet. Adding two tapers reduced transmission to 73.0 ± 1.0%, indicating that each taper introduced approximately 7.4% loss. Incorporating S-bends further decreased transmission to 53.3 ± 0.6%, corresponding to an additional 9.9% loss per S-bend. Maintaining such low losses enabled us to demonstrate a splitter with an added triangular cross-section MMI, achieving an unprecedented transmission above 50%. Due to the complexity of the measurements that include the multi-core fiber, we refer readers to [18] for further details on the results for the 1 × 4 splitter shown in Figure 10d–f. In addition to low losses, the results for the building blocks, including the S-bend, exhibit exceptional fabrication repeatability, as evidenced by standard deviations of 1% or less.

## 4. Discussion

Waveguide bends are essential components for achieving compact routing in integrated photonic circuits, and this holds equally true for 3D polymer platforms fabricated via two-photon polymerization (2PP). Despite their importance, relatively little attention has been devoted to the systematic study and optimization of individual building blocks in such 3D-printed platforms. Consequently, the literature offers limited guidance on design considerations, performance limits, and experimental feasibility of these components. This gap is particularly critical in 3D-printed photonics, where minimizing device footprint is often essential, and where the fabrication time and effort scale directly with the size and volume of the printed structure.

The high index contrast of air-clad 3D-printed waveguides often makes them highly multimode, which complicates the realization of low-loss bends when propagation in the fundamental mode is required. For IP-DIP polymer waveguides with a refractive index of 1.53 at 1550 nm, achieving single-mode guidance would require cross-sections close to 1 × 1 µm^2^. Such dimensions approach the resolution limit of the two-photon polymerization process and would lead to increased propagation losses due to surface roughness. On the other hand, waveguides mode-matched to standard telecom fibers, such as those with 14 × 14 µm^2^ cross-sections, are highly multimoded and unsuitable for sharp bends because the fundamental mode couples to higher-order modes even for moderate bends, making propagation of the fundamental mode prone to losses. As a practical compromise, we considered few-mode waveguides with widths of 2–4 µm while keeping the height fixed at 14 µm, since reducing a single dimension is sufficient when bending in one plane is considered. Reducing the width increases the separation between the effective indices of adjacent modes, which suppresses intermodal coupling and improves bend performance. Note that although the simulation results presented here primarily pertain to waveguides with a fixed height of 14 µm, the main conclusions and overall trends remain consistent when smaller waveguide dimensions are considered.

Our simulation results for 90° bends show that compact waveguide bends with spans as small as 10–30 µm can achieve near-unity transmission of the fundamental mode. This is especially true for the 2 µm wide waveguides, for which we identified that adiabatic transmission is possible with low Bézier parameters across the bend spans considered in the study. In contrast, the 3 µm and 4 µm wide waveguides require more precise tuning of Bézier and span parameters to maintain high transmission, as achieving low-loss performance for bends below 25 µm remains particularly challenging. These wider waveguides also exhibit multimode interactions at tighter bends, as higher-order modes are excited during propagation, which is evident from localized high-intensity regions in the bend. Conversely, the 2 µm wide waveguides demonstrate propagation closely resembling single-mode behavior, with minimal excitation of higher-order modes.

S-bends exhibit similar trends. For a 90 µm lateral offset that we considered here, aspect ratios below 1 are achievable, allowing highly compact layouts. Again, smaller waveguides and lower Bézier parameters yield the best performance, with the 2 µm wide waveguides offering the broadest design window and the least excitation of higher-order modes. Polarization dependence becomes more pronounced for narrower waveguides and sharper bends, so designs should be chosen to minimize differences between the TE and TM transmission.

Experimental demonstrations confirm that these design principles can be implemented in practice, underscoring the flexibility of the approach. However, additional experimental optimization beyond modeling is advisable to identify fabrication-tolerant configurations. To further reduce losses from an experimental perspective, it is essential to consider the primary loss mechanisms. Beyond the material losses associated with bulk absorption of IP-DIP, significant losses arise from waveguide wall roughness caused by discretization. We have observed that reducing the discretization step size mitigates these losses; however, they remain inherent to the layer-by-layer and line-by-line printing approach. Bends are typically more sensitive to such losses, as sharper geometries tend to push the mode toward the sidewalls, increasing roughness-induced attenuation. In our recent publication [41], we demonstrated that stitching introduces substantial losses once the printing area exceeds the objective’s field of view, which occurs, in our case, for components longer than 200 μm. Stitching accuracy is limited by translation stage positioning and alignment errors.

Compact, low-loss bends have been essential for realizing ultracompact and first-of-its-kind 3D splitters for multi-core fiber interconnects, demonstrating the practical impact of these design strategies. A deeper understanding of component performance, such as presented here, forms the backbone of such successful implementations. Overall, this work establishes practical design rules for low-loss bends in 3D polymer photonic circuits. These guidelines can significantly accelerate the development of complex architectures by reducing trial-and-error in layout design.

## 5. Conclusions

In this work, we presented a detailed numerical investigation of waveguide bends and established a practical workflow for designing such bends for 3D-printed photonic circuits. For air-cladded polymer waveguides fabricated via two-photon polymerization, achieving single-mode propagation is challenging because the required dimensions approach the printing resolution limit. Therefore, we focused on few-mode waveguides with widths of 2–4 µm, which represent a practical compromise between fabrication feasibility and optical performance. Our simulations show that for 90° bends, spans as small as 20–30 µm can theoretically achieve near-unity transmission of the fundamental mode when using 2 µm wide waveguides and low Bézier parameters. For wider waveguides (3–4 µm), maintaining high transmission requires careful tuning of bend span and curvature, as multimode interactions become significant. Similarly, S-bends, as demonstrated for designs with a 90 µm and 32 µm lateral offset, can achieve aspect ratios close to 1, enabling highly compact layouts. These results confirm that Bézier-shaped bends are advantageous due to their simplicity, smooth curvature profiles, and compatibility with design libraries in commercial tools such as Lumerical.

Although the study was conducted primarily in 2D, the design principles are equally applicable to 3D bends that maintain curvature within a plane. We demonstrated this by designing and fabricating S-bends for a 1 × 4 splitter, where 2D-optimized bends were first validated through 3D simulations and then implemented in full 3D geometry. Experimental results confirmed low-loss performance, with measured transmission exceeding 50% for the complete splitter and standard deviations below 1% across building blocks, highlighting excellent fabrication repeatability. It is important to note that this overall loss includes additional attenuation introduced by the S-bends. Each S-bend, designed for a 32 µm lateral offset in circular waveguides with a 2 µm diameter and a length of about 36 µm, contributed approximately 9.9% loss in our building block analysis.

Such systematic studies are essential for establishing design rules for the building blocks of 3D-printed photonic circuits. By providing quantitative guidelines for bend design, this work lays the foundation for accelerating the development of complex 3D photonic architectures and reducing reliance on trial-and-error approaches.

## Data Availability

The data presented in this study are available upon request from the corresponding author.
